# Supplementary material for: The causal relationship between the human gut microbiota and pyogenic arthritis: a Mendelian randomization study
Source: Front Cell Infect Microbiol. 2024 Nov 26;14:1452480. doi: 10.3389/fcimb.2024.1452480 (PMC11629706; doi:10.3389/fcimb.2024.1452480)
Supplement: Supplementary file 1 [file DataSheet1.zip › mendelian test/ebi-a-GCST90027505.csv_forest.pdf]

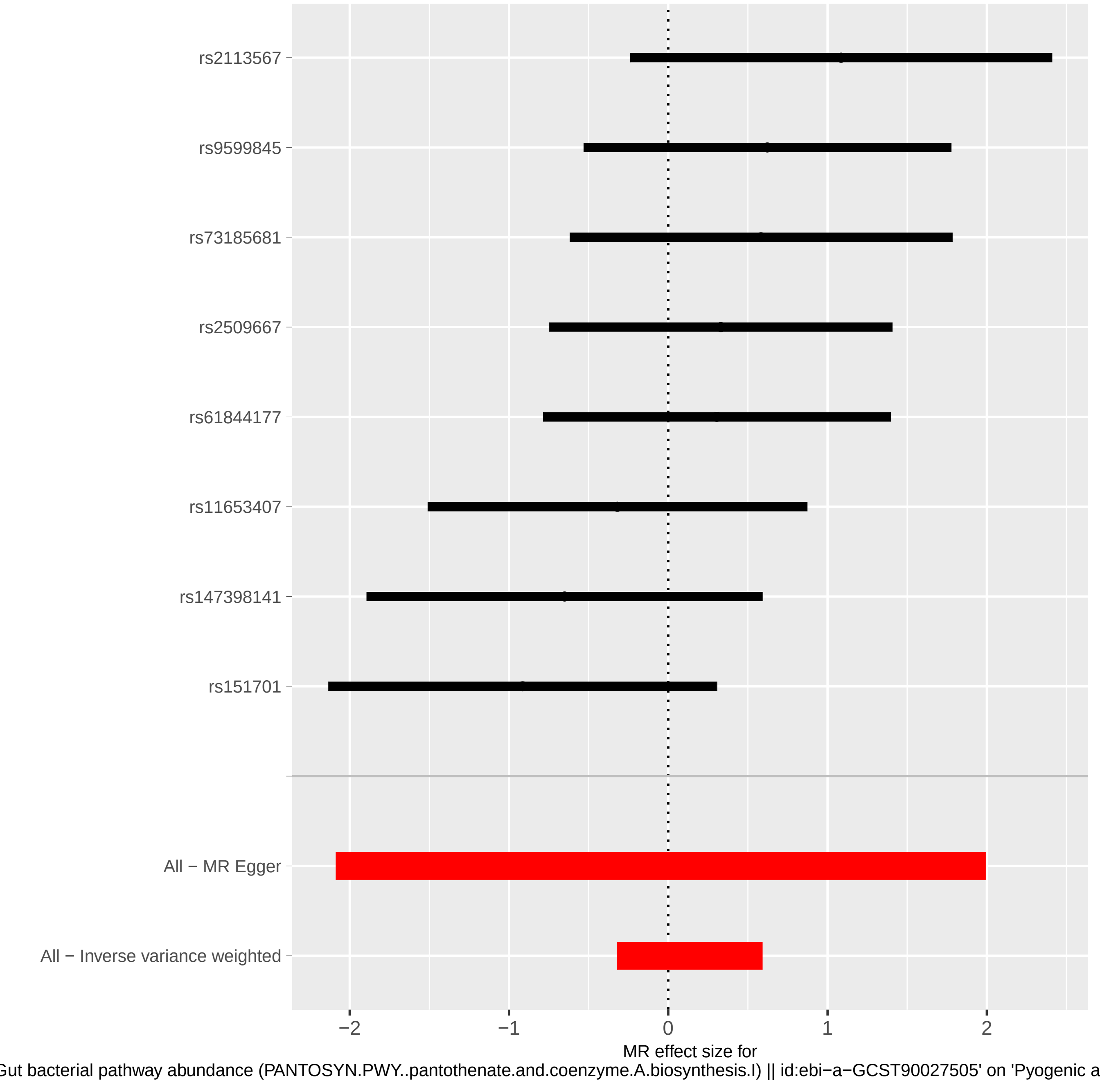
rs2113567
rs9599845
rs73185681
rs2509667
rs61844177
rs11653407
rs147398141
rs151701
All − MR Egger
All − Inverse variance weighted
−2
−1
0
1
2
MR effect size for
Gut bacterial pathway abundance (PANTOSYN.PWY..pantothenate.and.coenzyme.A.biosynthesis.I) || id:ebi−a−GCST90027505' on 'Pyogenic a
